# Bifunctional P-Containing RuO_2_ Catalysts Prepared from Surplus Ru Co-Ordination Complexes and Applied to Zn/Air Batteries

**DOI:** 10.3390/nano13010115

**Published:** 2022-12-26

**Authors:** Sebastián Lorca, Javier Torres, José L. Serrano, José Pérez, José Abad, Florencio Santos, Antonio J. Fernández Romero

**Affiliations:** 1 Grupo de Materiales Avanzados para la Producción y Almacenamiento de Energía, Universidad Politécnica de Cartagena, Aulario II, Campus de Alfonso XIII, 30203 Cartagena, Spain; 2 Departamento de Ingeniería Química, Área de Química Inorgánica, Universidad Politécnica de Cartagena, Plaza del Hospital 1, 30203 Cartagena, Spain

**Keywords:** Ru complexes, catalytic activity, ORR, OER, zinc/air batteries

## Abstract

An innovative synthetic route that involves the thermal treatment of selected Ru co−ordination complexes was used to prepare RuO_2_-based materials with catalytic activity for oxygen reduction (ORR) and oxygen evolution (OER) reactions. Extensive characterization confirmed the presence of Ru metal and RuP_3_O_9_ in the materials, with an improved electrocatalytic performance obtained from calcinated [(RuCl_2_(PPh_3_)_3_]. A mechanistic approach for the obtention of such singular blends and for the synergetic contribution of these three species to electrocatalysis is suggested. Catalysts added to carbon−based electrodes were also tested in all−solid and flooded alkaline Zn/air batteries. The former displayed a specific discharge capacity of 10.5 A h g^−1^ at 250 mA g^−1^ and a power density of 4.4 kW kg^−1^ cm^−2^. Besides, more than 800 discharge/charge cycles were reached in the flooded alkaline Zn/air battery

## 1. Introduction

Sustainable energy storage and conversion have lately received significant interest from academia and industry since it has emerged as the fundamental issue for maintaining stability in the new energy distribution grids. In addition, the need for high−energy−density storage technologies has caused the research community to turn away from the long-dominant Li-ion technology batteries to other ones that are more innovative and safer [1]. In this sense, rechargeable metal/air batteries are considered to be the upcoming technology for sustainable energy storage. Among other advantages, the use of inexpensive Zn as a negative electrode in metal–air batteries provides availability, the possibility of being used with aqueous electrolytes, and the well-known metal’s safety features [2]. Thus, Zn/air batteries could be considered good candidates to replace Li-ion batteries [3], although reliable rechargeable Zn/air batteries must be obtained first. Thus, several problems of the Zn electrode have to be addressed: namely, nonuniform Zn dissolution and deposition from/on negative electrodes, with electrode corrosion giving off H_2_ and significant overpotential during charging. Moreover, the long-standing difficulty for rechargeable Zn/air batteries is to achieve effective and reliable bifunctional catalysts for air electrodes [4], i.e., the oxygen reduction and evolution processes (ORR and OER) should have high efficiency, stability, and cyclability. Nevertheless, a good ORR catalyst is not always effective for OER, and vice versa; therefore, we see the necessity of studying both reactions independently.

The kinetics of the ORRs is one of the decisive factors in such devices [5]. Furthermore, many research groups have spent decades searching for reliable, effective catalysts to improve kinetics and reduce energy losses. To date, high proportions of the PGM (platinum group metals), such as Ru, Pt, or Pd have yielded the greatest results for catalytic efficiency and long-term stability. Despite this, the cost of PGM catalysts for massive implementation is economically prohibitive, except for valuable applications. In addition to price, the PGM group metals were regarded by the European Commission as critical materials in their initial report in 2011 [6]. Therefore, new strategies have been investigated to either replace or reduce the use of noble metals for ORR and OER catalysts [7]. Nowadays, great efforts have been carried out in two ways: the development of new metal-free catalysts based on N, B, or S-doped carbon materials [8,9], and the use of metal oxide catalysts, such as MnO_2_, RuO_2_, IrO_2_, Co_3_O_4_, or Cr_2_O_3_, greatly decreasing the amount of metal included in the air electrode [10,11,12,13,14,15,16,17]. Besides, metal oxide catalysts have been doped with several heteroatoms, such as P, S, or N to enhance their catalyst properties [18,19].

Although ruthenium metal shows catalytic activity towards ORRs [20], RuO_2_ has been one of the most explored transition metal oxides (TMOs) due to its high conductivity and excellent electrochemical reversibility for supercapacitor applications [21]. Furthermore, in recent years, RuO_2_-based positive electrodes have been employed for Li-ion [11] and vanadium redox flow [13] batteries, and they have been found to exhibit electrocatalytic properties in a variety of electrochemical processes: obtaining H_2_ from biomass [22], the photocatalytic oxidation of CO [12], or water oxidation [23]. Moreover, RuO_2_ has revealed very good catalytic behavior towards OERs and ORRs, thus proving it has excellent bifunctional properties for Zn/air batteries [14].

It has been claimed that both the synthetic method and the precursor of choice have a strong influence on the final performance of rutile-type RuO_2_ catalysts in terms of stability and electrocatalytic activity for the water splitting reaction. In fact, it has already been revealed that the synthesis procedure clearly influences the obtained RuO_2_ [24,25,26], although just a few recent examples have used Ru(II) and Ru(III) complexes as precursors [27,28]. In this sense, we have recently reported the use of Pd and Ru excess in surplus coordination/organometallic complexes prepared in research laboratories [29,30] as precursors for Pd/PdO and Ru/RuO_2_ materials. In this work, we integrate this oriented, precious metal recovery into our approach toward RuO_2_-based catalyst materials for OERs and ORRs, which are eventually incorporated into Zn/air batteries. The unexplored relationship between the type of ligands coordinating Ru in the complexes and the final composition/catalytic properties of the RuO_2_-based materials obtained by its calcination is also presented here.

In this regard, international regulations encourage the recycling of noble and precious metals in order to protect the environment and avoid the depletion of natural resources [29,30,31]. This new trend, with its corresponding enforcing laws, has adopted the term “urban mining”, which is used to describe the process of recovering raw materials from spent products, buildings, and waste [32]. This paper combines a multidisciplinary approach to devise new bifunctional catalysts for ORRs and OERs. The entire process means a route to synthesize (from molecular precursors) unique heterogeneous catalysts that can be used in Zn/air batteries. Furthermore, this process implies managing wastes in an environmentally friendly way and taking advantage of the secondary materials that they contain, as stated in EU environmental policy [33]. These valuable catalysts were integrated into air electrodes, and their performance was correlated with their physicochemical and electrochemical properties, confirming, in this way, the real application of our materials in advanced storage energy devices.

## 2. Materials and Methods

### 2.1. Chemicals

Zinc powder (<10 µm, >98%), KOH (85%), PVA MOWIOL 18–88 (MW 130.000), [RuCl_3_∙xH_2_O], [RuCl_2_(p−cymene)]_2_, [(RuCl_2_(PPh_3_)_3_], and commercial RuO_2_ were obtained from Merck Life Science S.L.U. (Madrid, Spain). Carbon black was purchased from Alfa Aesar (Thermo Fisher GmbH—Kandel, Germany).

### 2.2. Synthesis Methods

**Scheme 1:**
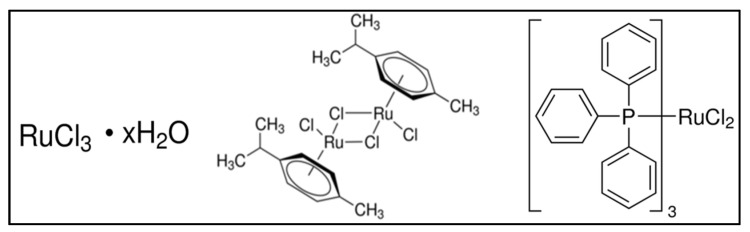
Structures of the catalyst precursors or starting material. Left: [RuCl_3_∙xH_2_O]; middle: [RuCl_2_(p−cymene)]_2_; right: [RuCl_2_(PPh_3_)_3_].

The synthesis of ruthenium (IV) oxide (RuO_2_) is usually carried out by heat treatment of RuCl_3_ [15]; however, in this work, different Ru complexes were used in addition to RuCl_3_. Thus, to prepare the catalysts, we started with three Ru compounds: hydrated ruthenium trichloride [RuCl_3_∙xH_2_O], dichloro(p−cymene) ruthenium(II) dimer [RuCl_2_(p−cymene)]_2_, and (Tris(triphenylphosphine) dichlororutenium [(RuCl_2_(PPh_3_)_3_] (Scheme 1).

Catalysts were synthesized by thermal treatment of the precursor compounds. A total of 200 mg of each compound, [RuCl_3_∙xH_2_O], [RuCl_2_(p−cymene)]_2_, and [RuCl_2_(PPh_3_)_3_], was calcined separately for 3 h at 900 °C in static air using a Thermo Scientific Heraeus muffle furnace (Thermo Fisher GmbH—Kandel, Germany) and alumina crucibles. Similarly, another two crucibles containing only [RuCl_2_(PPh_3_)_3_] were calcined at 500 and 700 °C for the same time and under the same conditions. When the [RuCl_3_∙xH_2_O] and [RuCl_2_(p−cymene)]_2_ complexes were calcined at 900 °C, this produced samples of RuO_2_ only, which are hereinafter referred to as RuO_2_ [RuCl_3_] and RuO_2_ [Cym], respectively. Although only RuO_2_ is also obtained when calcining these compounds at much lower temperatures, knowing that the catalytic properties may change with the calcination temperature [17], 900 °C was chosen for all precursors to better compare them with the catalyst obtained from [RuCl_2_(PPh_3_)_3_]. The calcination product obtained from the latter is very different from that found using the [RuCl_3_∙xH_2_O] or [RuCl_2_(p−cymene)]_2_ complexes. In this case, at lower temperatures, some Ru metal is also observed, resulting an xRu(1−x)RuO_2_ material. By adjusting the temperature, it is possible to vary the value of x, which decreases with temperature [30]. Besides, [RuCl_2_(PPh_3_)_3_] calcination at 900 °C produced Ru/RuO_2_, but also a residual amount of phosphorous was detected, which has been identified by XRD with the formation of RuP_3_O_9_ [30]. In this work, this catalyst has been named RuO_2_ [PPh_3_] 900 °C. To evaluate the variation in the phosphorous content with temperature, the same precursor was also calcined at two different temperatures (500 °C and 700 °C) under the same conditions used before.

Polyvinyl alcohol (PVA) gel, which was used as an electrolyte in galvanostatic discharges and polarization tests, was fabricated by a casting solution method, as was previously reported [34].

### 2.3. Structural Characterization

X-ray photoemission spectroscopy (XPS) spectra were taken using a PHOIBOS 150 2D−C and Monochromatized Al Kα (1486.7 eV) X-rays. Scan spectra were obtained at an analyzer pass energy of 10 eV, which provided a resolution of 0.58 eV, measured at Ag 3d_5/2_ core level. The contribution of the backgrounds was eliminated by a Shirley routine, before the spectra were analyzed. The fitting of the Ru 3d and O 1s peaks was carried out using asymmetric Lorentzian–Gaussian convoluted curves, with a spin−orbit splitting of 4.2 eV and an intensity ratio of around 0.67 for the Ru 3d doublet, in agreement with the literature [35]. Peaks for all other core levels are fitted using a Gaussian (70%)–Lorentzian (30%) blends.

A Hitachi S−3500N scanning electron microscope (Hitachi High−Technologies Corporation, Tokyo, Japan) was used to obtain the scan electron microscopy (SEM) images, which utilize 70−chamber pressure for BSE or <1 Pa for SE. For energy dispersive X-ray spectroscopy (EDX) analyses, an XFlash 5010 Bruker AXS Microanalysis was utilized (Bruker Española S.L; Rivas−Vaciamadrid, Spain), with a resolution of 129 eV. Two different samples were analyzed: catalyst powders and Pt plates coated with samples in Nafion/isopropanol solution, which were let to dry overnight. 

X-ray diffractograms were recorded with a Bruker D8 Advance diffractometer. The 2θ range was 10–70°, with a step of 0.05°; Cu Kα radiation was used. 

TG analysis was carried out in a TGA/DSC 1HT Mettler−Toledo thermobalance in air atmosphere using a crucible made of alumina. Gram scale calcinations to obtain the catalysts were performed in a Thermo Scientific Heraeus M110 muffle.

### 2.4. Electrochemical Characterization

A rotating disk−ring electrode system, RRDE−3A (Als Co. Ltd.; Tokyo, Japan), with a glassy carbon (GC) disk and a Pt ring, was used accoupled to a Biologic VSP Modular 5 channels potentiostat/galvanostat. Besides, a Hg/HgO and a GC bar were used as reference and counter electrodes, respectively. The glassy carbon disk electrode with a 0.1256 cm^2^ surface area (4 mm in diameter) was polished to a mirror using various alumina grades. With the aim of obtaining a reproducible electrode surface before adding the catalyst inks, the disk and ring of the RDEE were cycled in an N_2_−saturated solution of HClO_4_ 0.1 M, following the method proposed by Garsany et al. [36].

Inks of different Ru−based materials were prepared by adding 12 mg of each Ru−based material to 2 mL of 5% Nafion/isopropanol solution. To add the inks to the GC disk, this electrode was turned upright, and then 10 µL of the resulting inks were pipetted onto the electrode. After that, in order to completely dry the ink, the electrode was rotated at an increasing rotating rate until a homogeneous film was obtained.

Afterward, the RRDE was cycled at a scan rate of 10 mV s^−1^ between 0.2 and −1 V vs. Hg/HgO in an N_2_−saturated 0.1 M KOH solution at room temperature until repetitive voltametric cycles resulted. Next, the solution was saturated with O_2_ gas during 10 min and the linear sweep voltammetry (LSV) curves were obtained between 0.2 and −1 V vs. Hg/HgO at a scan rate of 10 mV s^−1^. A constant potential of 1.4 V vs. RHE was applied to the ring electrode during the experiments. 

Catalysts were used to prepare the positive electrodes, which were tested in a Zn/PVA−KOH/air battery, including 1 g of Zn powder anode and a PVA−KOH gel polymer electrolyte (GPE) with a volume of 0.51 cm^3^, which was synthesized as described previously [34]. Nickel meshes were used as current collectors, and the electrode/GPE contact areas were always 1.1 cm^2^. The same Biologic potentiostat/galvanostat was used to perform the battery tests. Galvanostatic discharge curves were carried out at −10 mA.

To prepare the cathode electrodes for discharge and polarization measurements, 100 µL of each product ink was pipetted on top of a 1.1 cm^2^ disk, obtained by pressing nickel mess, carbon black, and polyvinylidene difluoride (PVdF) as binder (5% wt.) in a hydraulic press at 250 bars during 1 min. Total weights of the catalysts in the air electrode were 0.015 ± 0.005 g. For the flooded Zn/air Batteries, 15 µL of the ink was added to a paper carbon purchased from Sigracet, and ~1 mL of 8M KOH solution was used.

## 3. Results

### 3.1. Structural Characterization

#### 3.1.1. EDX and SEM

**Figure 1:**
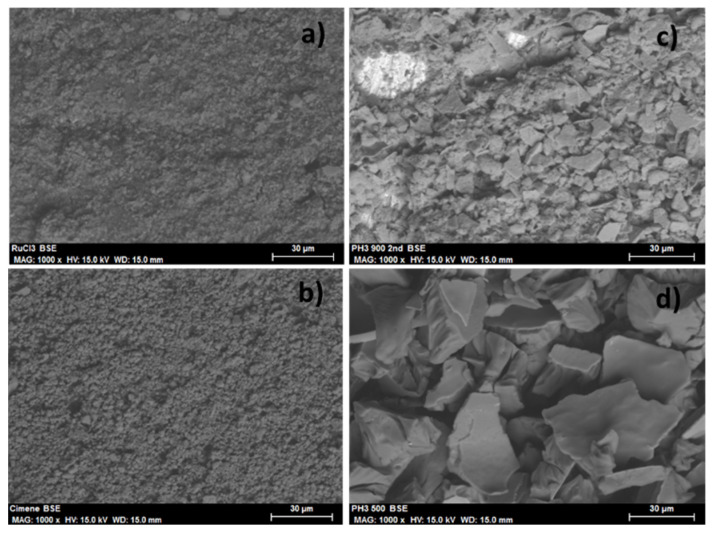
Scanning electron microscope (SEM) images of the catalyst inks deposited onto Pt: (**a**) RuO_2_ [RuCl_3_], (**b**) RuO_2_ [Cym], and (**c**) RuO_2_ [PPh_3_] calcined at 900 °C; (**d**) RuO_2_ [PPh_3_] obtained at 500 °C.

In order to analyze the real morphological structure of the catalyst materials as they are deposited onto the RRDE electrode for electrochemical analysis, the Pt plates were coated with the Nafion/isopropanol inks, and SEM images of each material were captured. Figure 1a,b depict minor structural variations between RuO_2_ [RuCl_3_] and RuO_2_ [Cym]. However, laminar forms are seen for the RuO_2_ [PPh_3_] obtained at 900 °C (Figure 1c), which changes significantly from the spherical ones seen in the other two materials.

**Figure 2:**
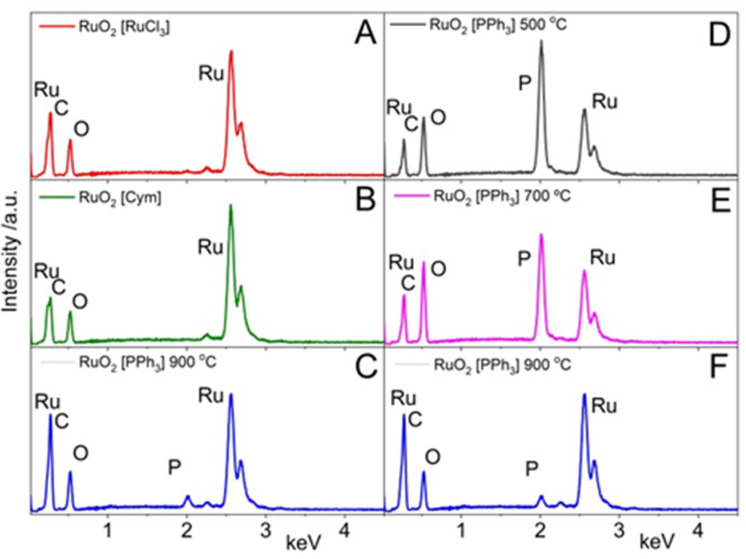
Energy Dispersive X-ray (EDX) spectra of the calcined materials obtained at 900 °C in static air: (**A**) RuO_2_ [RuCl_3_]; (**B**) RuO_2_ [Cym]; (**C**) RuO_2_ [PPh_3_] and EDX spectra of calcined [RuCl_2_(PPh_3_)_3_] at three different temperatures: (**D**) 500 °C, (**E**) 700 °C, and (**F**) 900 °C in the same conditions.

Besides, the EDX spectra of the three powders of RuO_2_ [RuCl_3_], RuO_2_ [Cym], and RuO_2_ [PPh_3_] of the materials produced by calcination at 900 °C are shown in Figure 2A–C, respectively. The only peaks observed for the RuO_2_ [RuCl_3_] and RuO_2_ [Cym] materials are those associated with Ru and O, indicating that their composition is mostly RuO_2_. Both peaks can also be observed in Figure 2C, albeit there is also a minor peak for P, showing that some of the phosphorus from the starting material, [RuCl_2_(PPh_3_)_3_], still remains in the obtained product after calcination. This result agrees with the XRD study described below in this work and also with the one carried out previously by us, where the presence of RuP_3_O_9_ was detected after the calcination of the [RuCl_2_(PPh_3_)_3_] complex [30].

These differences observed for RuO_2_ [PPh_3_], with respect to those obtained from the RuO_2_ [RuCl_3_] and RuO_2_ [Cym] materials obtained at 900 °C, led us to calcinate the initial complex [RuCl_2_(PPh_3_)_3_] at 500 and 700 °C with the aim of studying the composition and morphology of the materials obtained at lower temperatures. Thus, the EDX analysis shows more intense peaks of P for the [RuCl_2_(PPh_3_)_3_] calcined at 500 and 700 °C; the intensity decreases with increments in the calcination temperature, as displayed in Figure 2D–F and Table S1. Besides, the SEM images demonstrate a morphological change with calcination temperature. Figure 1D shows that RuO_2_ [PPh_3_] 500 °C presents, again, a laminar structure but with greater plates than those observed for the materials calcinated at 900 °C. Figure 2 further demonstrates that all Cl atoms from the precursor compounds have been removed.

**Figure 3:**
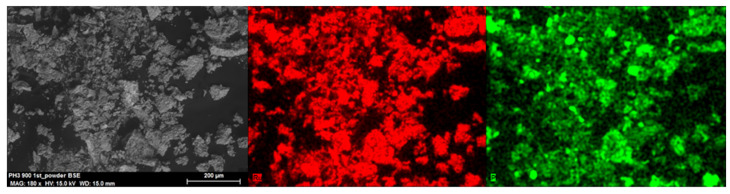
SEM image of the RuO_2_ [(PPh_3_)] powder obtained by calcination at 900 °C in static air and the corresponding EDX mapping to evidence the Ru (red) and P (green) distribution.

Finally, SEM imaging and EDX mapping were carried out to verify the homogeneous distributions of Ru and P inside the RuO_2_ [PPh_3_] material obtained at 900 °C. As shown in Figure 3, the P atoms are localized where Ru is also observed, which is in agreement with the formation of the RuP_3_O_9_ proposed previously.

Additional SEM and EDX measurements for the RuO_2_ [PPh_3_] obtained at different temperatures and supported on the Pt plates are included in Figure S1. The mapping images of the 500 °C−calcinated material evidence that many of the lamellar structures are composed mainly of Ru metal (note the deeper red color). However, as the temperature increases, the tone becomes more magenta due to the presence of oxygen (blue color), which points to an oxidation process from Ru metal to RuO_2_, as is confirmed below by the XRD analysis.

#### 3.1.2. XRD

**Figure 4:**
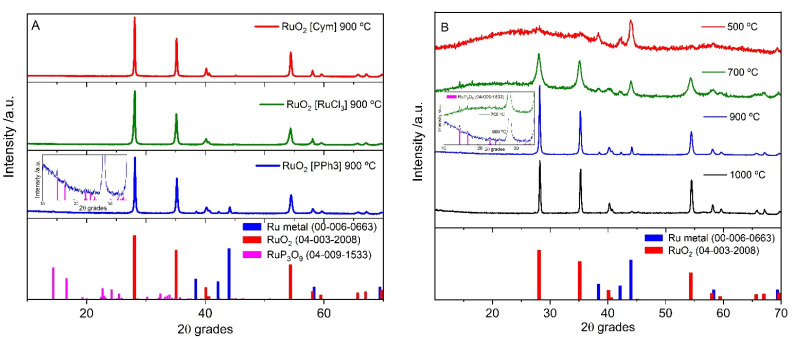
(**A**) XRD patterns of the calcined materials obtained at 900 °C: RuO_2_ [RuCl_3_], RuO_2_ [Cym], and RuO_2_ [PPh_3_]. (**B**) XRD patterns of [RuCl_2_(PPh_3_)_3_] calcined at different temperatures: 500 °C, 700 °C, 900 °C, and 1000 °C.

The XRD patterns of the three Ru complexes calcinated at 900 °C are shown in Figure 4A. These results agree with those reported before (in [30]); only peaks corresponding to RuO_2_ were obtained for the RuO_2_ [RuCl_3_] and RuO_2_ [Cym] materials, while RuO_2_ [PPh_3_] also shows Ru metal and RuP_3_O_9_ peaks. The presence of RuP_3_O_9_ was previously reported by us using XRD and synchrotron radiation [30].

XRD patterns of the RuO_2_ [PPh_3_] obtained by thermal treatment at 500, 700, 900, and 1000 °C are also displayed in Figure 4B. The pattern of RuO_2_ [PPh_3_] at 500 °C presents intense peaks due to the Ru metal, while the RuO_2_ versions are very low. However, the intensity of the RuO_2_ peaks increases with calcination temperature, whereas this decreases the Ru metal peaks. In addition, RuP_3_O_9_ appeared in both the RuO_2_ [PPh_3_] 700 and 900 °C diffractograms (insets of Figure 4). Finally, when 1000 °C was reached, only RuO_2_ was observed in the RuO_2_ [PPh_3_] material, supporting the complete oxidation of the Ru metal to RuO_2_ when all phosphorous is decomposed, as discussed below. 

Indeed, these results can be explained when taking into account the TG analysis that was carried out previously by us, as described in reference [30] (also Figure S2 includes the TGA curve obtained again for [RuCl_2_(PPh_3_)_3_]). The TGA curves obtained for [RuCl_3_∙xH_2_O] and [RuCl_2_(p−cymene)]_2_ differ greatly from those recorded for [RuCl_2_(PPh_3_)_3_] [30]. During heating, the first two materials lose weight up to ~420 °C, where RuO_2_ is formed, and stable behavior is maintained till ~1200 °C. On the contrary, the decomposition of [RuCl_2_(PPh_3_)_3_] was not completed until 720 °C. Furthermore, the presence of Ru metal can be associated with the existence of P−donor ligand in the initial compound since P would favor the reduction of Ru(II) to Ru metal, retarding the formation of RuO_2_, as it is confirmed by the XRD included in Figure 4B. Only when higher temperatures are reached, and no phosphorous remains in the material is all the Ru metal oxidized to RuO_2_, as confirmed by the XRD diffractogram of [RuCl_2_(PPh_3_)_3_] calcinated at 1000 °C (Figure 4B).

#### 3.1.3. XPS

**Figure 5:**
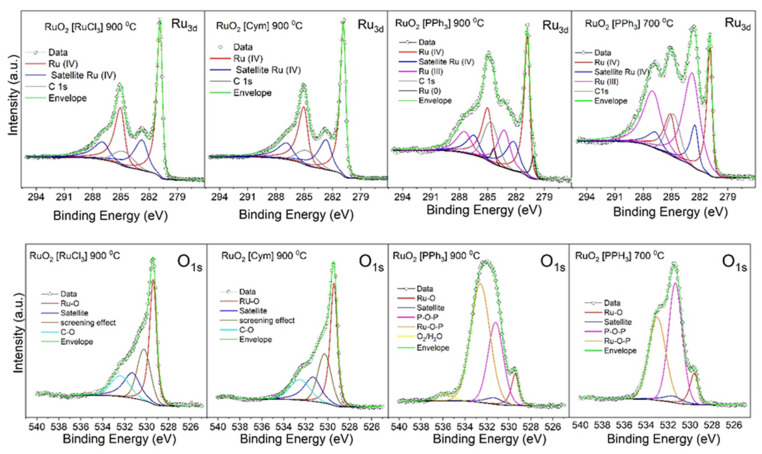
Ru3d and O1s spectra for the RuO_2_ [RuCl_3_], RuO_2_ [Cym], and RuO_2_ [PPh_3_] materials calcined at 900 °C and the RuO_2_ [PPh_3_] obtained at 700 °C.

X-ray photoelectron spectra of the three materials obtained by calcination at 900 °C, and that of RuO_2_ [PPh_3_] calcined at 700 °C, were also recorded to get insights into the structure and composition of the catalytic materials. Once again, very different behavior was found for the RuO_2_ [PPh_3_] materials with respect to RuO_2_ [RuCl_3_] and RuO_2_ [Cym] (Figure 5). The Ru 3d, Ru 3p, and O 1s peak regions for these catalysts match perfectly with the results reported frequently in the literature for RuO_2_ [35,37,38,39]. However, the XPS spectra obtained for the RuO_2_ [PPh_3_] at 700 and 900 °C present additional peaks in these regions, which must be related to the presence of RuP_3_O_9_ inside the calcined materials, as was confirmed by the XRD in this work and before [30]. The presence of P in these samples is confirmed by the P 2s peak found at 191.5 eV (Figure S3) due to the P bonded to O in (PO_3_)^−^ [40,41]. Besides, Figure 5 shows the deconvoluted Ru 3d regions of the XPS spectra obtained for the three materials analyzed, and the fitting parameters are shown in Table S2. It is important to note that there is an overlap in the Ru 3d region between the Ru 3d_3/2_ (285.0 eV) and C 1s (284.7 eV) core levels; therefore, the peak deconvolution process must be carried out carefully. As can be seen, the Ru 3d region peaks found for RuO_2_ [RuCl_3_] and RuO_2_ [Cym] are similar to those reported previously for RuO_2_: two components due to Ru(IV), Ru 3d_5/2_, and Ru 3d_3/2_ at 280.8 and 285.0 eV, with two satellites at 282.7 and 286.9 eV. The presence of these satellites was studied in detail in the literature [35]_._ However, two additional peaks at 283.3 and 287.5 eV are observed for RuO_2_ [PPh_3_] at 900 °C and at 282.8 and 287.0 eV for the material studied at 700 °C. The new peaks have been assigned to Ru(III), which is in agreement with the formation of RuP_3_O_9_ since this compound presents the typical structure of metaphosphates: Ru (PO_3_)_3_ [42,43]. This result also agrees with others reported previously, where these peaks were assigned to Ru(III) species [35,38]. Note also the appearance of a peak at 280.2 eV in the RuO_2_ [PPh_3_] at 900 °C, corroborating the presence of Ru metal. Moreover, Figure S4 shows the Ru 3p region, confirming again the different behavior observed for RuO_2_ [PPh_3_] with respect to RuO_2_ [RuCl_3_] and RuO_2_ [Cym].

Regarding the O 1s region displayed by the RuO_2_ [RuCl_3_] and RuO_2_ [Cym] catalysts, this closely resembles the results reported frequently in the literature for RuO_2_. The main component located at 529.4 eV corresponds to O bonded to Ru, while the second component at 531.3 eV is associated with the Ru 3d satellite peak. The component at 530.3 eV has been tentatively assigned in the literature to surface hidroxide. However, it has also been proposed that this feature is mainly due to the many body screening effect of the conduction electrons, as it has been observed in other rutile oxides, such as IrO_2_ [35]. In the literature, the last component at 532.4–532.5 eV has been assigned to organic C–O bonds since the fitting of the Ru 3d region indicates the presence of C contamination located at 284.7 eV; therefore, the presence of this O component is plausible. The O 1s spectra for the RuO_2_ [PPh_3_] at 900 °C and 700 °C are completely different from the RuO_2_ [RuCl_3_] and RuO_2_ [Cym] materials. The lattice O bonded to Ru, and the satellite components (maintaining the O–Ru/satellite ratio as in the Ru 3d core level) are also present, but they are not the dominant features. For the RuO_2_ [PPh_3_] at 900 °C, three new components appear, located at 531.2 eV, 532.6 eV, and 535.8 eV. The first component is assigned to the O bonded to two P (P–O–P), while the second one is assigned to the O bonded to P and Ru (Ru–O–P) in the ruthenium metaphosphate structure, where the oxygen Ru−O−P atoms are double that of P–O–P, which is in good agreement with the area ratio of both components (~1.8). The last and minor component at 535.8 eV is assigned to the O_2_ and/or H_2_O adsorbed on the surface [44]. Interestingly, for the RuO_2_ [PPh_3_] sample at 700 °C, both the P–O–P and Ru–O–P components are present at 531.3 eV and 533.0 eV, respectively. However, the P–O–P component is higher than the Ru–O–P, with a (Ru–O–P)/(P–O–P) area ratio of ~0.9, as an indication that the ruthenium metaphosphate structure at 700 °C was not completely formed.

### 3.2. Electrochemical Measurements

#### 3.2.1. ORR Activity of the Different Materials

The electrocatalytic performance of our RuO_2_−based materials was tested in a 0.1 M KOH solution because KOH is the most common electrolyte for metal–air batteries [45]. The electrocatalytic activity of the different materials towards the ORR can be compared on the basis of different parameters, such as the diffusion−limited current density (JD), the kinetic current density (JK), the onset potential, and the number of exchanged electrons (n). These parameters are determined from the LSV curves obtained using a rotating ring-disk electrode. In this work, two or three complete experiments for each material were carried out, both for the ORR and OER measurements. The values included in Table 1 and Table 2 were calculated by obtaining the average of the different results carried out with each catalyst. The errors are also indicated. 

**Figure 6:**
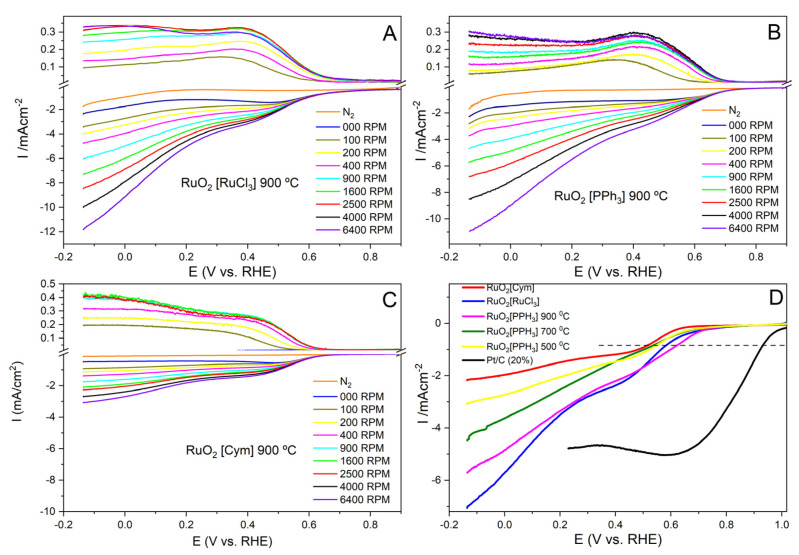
LSV curves of the explored materials: (**A**) RuO_2_ [Cym], (**B**) RuO_2_ [RuCl_3_], and (**C**) RuO_2_ [PPh_3_] calcined at 900 °C, respectively, obtained using an RRDE in O_2_ and N_2_−saturated 0.1 KOH. Rotation rate values are included in each graph. (**D**) ORR LSV curves of the different materials at 1600 rpm compared with Pt/C (20%) curve.

As can be seen in Figure 6 and Figure S5, the LSV curves obtained in the N_2_−saturated electrolyte do not show a peak, but in the oxygen−saturated solution, an increase in cathodic current was observed. This fact is attributed to the catalytic activity (for ORR) of the materials placed on the disk electrode. Besides, the increase in disk current intensity at the same potential observed with faster rotation speeds indicates a convective mass transport influence. In Figure 6D, the LSV curves of the different materials obtained at 1600 rpm are compared in the range of 0.35–0.9 V vs. RHE. The more positive onset corresponds to the RuO_2_ [PPh_3_] at 900 °C, followed by RuO_2_ [RuCl_3_], reaching 0.603 and 0.565 V vs. RHE at a current density of −1 mA cm^−2^, respectively (Table 1).

Additionally, Figure S6 compares the cathodic LSV curves recorded for the commercial RuO_2_ and the RuO_2_ [PPh_3_] at 900 °C, confirming the best catalytic behavior for the ORR of the material obtained in this work. 

**Figure 7:**
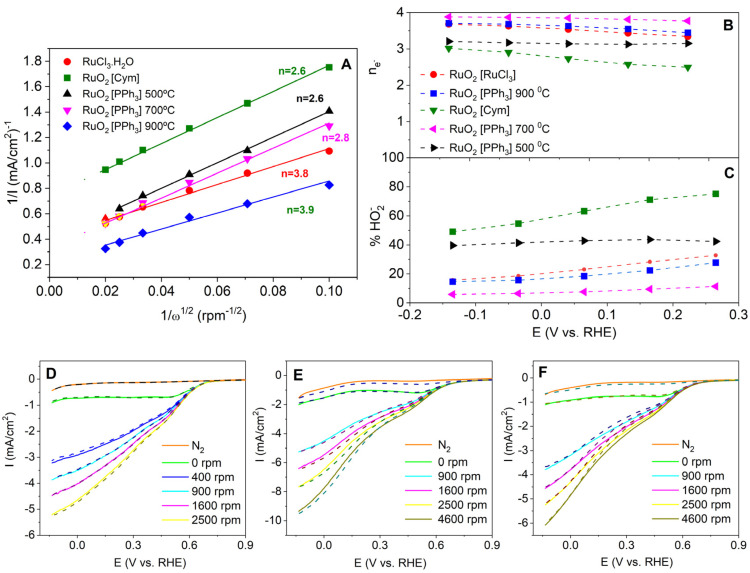
(**A**) KL plots for the different materials at −0.6 V (vs. Hg/HgO), including the n value obtained at this potential. (**B**) Dependence of the electron transfer number and (**C**) the HO_2_^−^ values with electrode potential at 1600 rpm from data in Figure 5 using Equations (S.3) and (S.4). A complete set of plots showing the KL plots, n, and the HO_2_^−^ values at different potentials for all materials can be found in the supplementary materials file (Figures S7 and S8). LSV results obtained before and after 1000 voltametric cycles (0.265–0.865 V vs. RHE) for (**D**) RuO_2_ [PPh_3_], (**E**) RuO_2_ [RuCl_3_] and (**F**) RuO_2_ [Cym] in an O_2_–saturated KOH 0.1 M solution at different rotations.

In order to obtain all the kinetic parameters for the ORR (Figure 7, Figures S7 and S8), a Koutecky–Levich (KL) expression (Equation (S2)) was first applied. However, the use of the KL method has raised many controversies because important factors, such as scan rate, the composition of the catalyst, and the preparation of the electrodes, influence the electrocatalytic results, which may lead to wrong KL treatment [46]. Besides, it should be noted that KL theory does not provide information about the mechanistic pathways of the process and that the KL expression was initially established to measure some physical quantities, such as the diffusion coefficient of solutes, using specific electrochemical reactions. Consequently, the electron transfer number, n, was contemplated as a constant in the KL method. Conversely, n is very sensitive to the applied potential and it does not maintain a constant value during the ORR process measured by RRDE; furthermore, this method would not be suitable to determine n for the ORR [47,48]. In any case, this method is still widely used to analyze the electrochemical kinetics of ORR electrocatalysts [49,50]. 

The inconsistencies arising when the electron transfer number was obtained by the KL method pointed out the noncompliance of the KL assumptions. Thus, to gain a deeper insight into the reaction kinetics, it is recommended that RRDE tests be used to obtain the electron transfer number and the percentage ratio of HO_2_^−^ by using Equations (S3) and (S4) [51].

Figure 7B,C depicts the results obtained using Equations (S.3) and (S.4) for the different materials deposited onto the disk electrode for a potential range between −1.0 to −0.6 V vs. Hg/HgO and a rotating rate of 1600 rpm. As can be seen, n maintains a quasiconstant value with respect to the voltage for all the materials analyzed. Besides, Figures S7 and S8 display the n and %HO_2_^−^ values for all the materials analyzed in this article at all rotating rates. 

The discrepancies between the results obtained using the Koutetecky–Levich method, or Equations (S.3) and (S.4), have often been reported. Furthermore, the KL limitations should be considered when complex kinetic processes are analyzed, as has been frequently established by different authors [46,52,53]. In order to summarize the results obtained during the analysis of the ORR process, Table 1 shows all the parameters that characterize the catalysis behavior of the materials used.

This table also includes the Tafel slopes measured for all catalysts (Figure S9), which give information about the mechanism of the ORR [54]. RuO_2_ [RuCl_3_] presents the maximum Tafel value obtained, pointing to a high overpotential for the ORR, whereas RuO_2_ [Cym] shows a lower Tafel value, but its onset and the LSV current density values indicate that it catalyzes the ORR poorly. Once again, the RuO_2_ [PPh_3_] materials obtained at the three different temperatures showed distinctive behavior with intermediate Tafel values. For the RuO_2_ [PPh_3_] catalysts obtained at 700 and 900 °C, the same Tafel slope resulted: 106 mV dec^−1^. A Tafel value close to 110 mV dec^−1^ has been frequently associated with the ORR mechanism, where the determining rate step corresponds to the first electron transference following the reaction [55]:

(1) $$*+{\;O}_{2}+{\;e}^{-}\to \;*-O_{2}^{-}$$

where * means an active site in the material where an O_2_ molecule is adsorbed. This fact suggests that the ORR for RuO_2_ [PPh_3_] follows an associative−type mechanism. Knowing that the n value resulting from the kinetic analysis is ~4 for RuO_2_ [PPh_3_] at 700 and 900 °C, and according to a previously reported ORR mechanism in an alkaline medium [56], the next reactions can be suggested to complete the ORR mechanism for RuO_2_ [PPh_3_] at 700 °C and 900 °C:

(2) $$*-O_{2}^{-}+H_{2}O+1e^{-}\to \;*-O+2{\mathrm{OH}}^{-}$$

(3) $$*-O\;+{\;H}_{2}O\;+1e^{-}\to \;*-\mathrm{OH}+{\mathrm{OH}}^{-}\;$$

(4) $$*-\mathrm{OH}\;+1e^{-}\to {\mathrm{OH}}^{-}+\;*\;$$

Moreover, the durability of the materials was evaluated in accelerated stability tests by cycling them in the potential range of −0.6–0.0 V vs. Hg/HgO in an O_2_−saturated 0.1 M KOH solution at a scan rate of v = 0.05 V s^−1^. The LSV curves (prior to and after 1000 cycles) were compared under both N_2_ and O_2_ conditions. Figure 7 displays the LSV obtained before and after 1000 voltametric cycles (0.265–0.865 V vs. RHE) for D: RuO_2_ [PPh_3_], E: RuO_2_ [RuCl_3_], and F: RuO_2_ [Cym] in O_2_−saturated KOH 0.1 M at different rotation rates, which demonstrates good stability in the materials obtained by calcination at 900 °C.

#### 3.2.2. QER Activity of the RuO_2_−Based Materials

One of the most critical issues regarding metal/air batteries is their non rechargeability, which is partly caused by the fact that most catalysts used in metal/air batteries only catalyze the ORR or the OER but not both reactions. Only a few materials that are able to act as bifunctional electrodes in metal/air batteries have been described, and none of them have been found to show enough catalytic activity to be used in a commercial battery. Among them, ruthenium oxide has been reported to work as an excellent electrode material for the OER [14,16,57]. Moreover, a recent work explored the reversibility of an electrode consisting of RuO_2_, supported on carbon nanofibers, with encouraging results [58]. Thus, we decided to find out if our materials could also act as catalysts for the OER process and to explore if phosphorus doping would influence RuO_2_ catalysis towards the OER.

**Figure 8:**
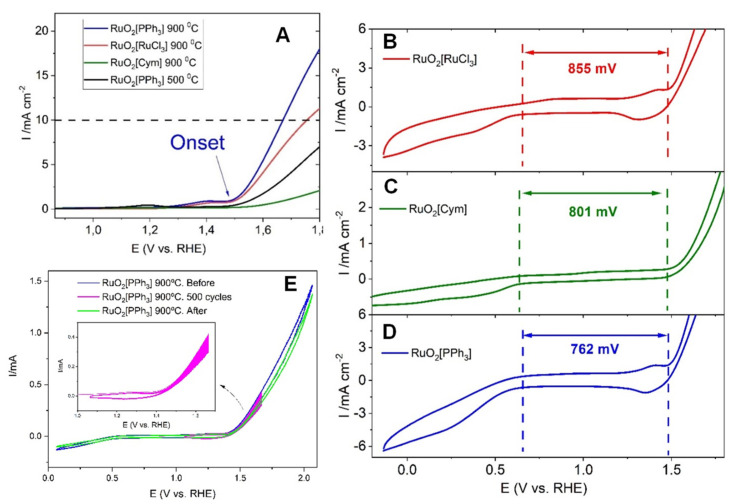
(**A**) LSV in the oxidation branch, showing the OER onset potential of each material in 0.1 M KOH. (**B**–**D**) CV, including the ORR and OER ranges for RuO_2_ [RuCl_3_], RuO_2_ [Cym], and RuO_2_ [PPh_3_] at 900 °C in 0.1 M KOH. Numbers indicate the ΔE_onset_(E_OER_ − E_ORR_) values included in Table 2. Dashed lines are included as eye guides. (**E**) CV obtained for RuO_2_ [PPh_3_] at 900 °C in 0.1 M KOH at 1600 rpm and scan rate of 100 mV s^−1^, demonstrating the stability of this material for ORR and OER processes.

The OER results for the three RuO_2_-based materials obtained from the starting compounds by calcination at 900 °C are shown in Figure 8 and Table 2. In this case, all the materials have a similar onset value, although RuO_2_ [Cym] presents the lowest one: 1498 mV vs. RHE; however, very low current densities discard this material from being a good catalyst for OERs. However, RuO_2_ [PPh_3_] exhibits higher current densities, reaching 10 mA cm^−2^ at 1670 mV vs. RHE. Besides, when E_ORR_ is used to get the ΔE_onset_ (E_OER_ − E_ORR_), RuO_2_ [PPh_3_] reported the smallest voltage gap: 762 mV vs. RHE when considering the E_OER_ and E_ORR_ onsets, and 1068 mV vs. RHE using the voltage values at −1.0 and 10 mA cm^−2^ current densities. Figure 7B–D compare the voltametric cycles, including the ORR and OER regions, for the three materials obtained at 900 °C. These results for the OER are in the same range as those previously reported for other groups [16,23,58].

**Table 2:** Summary of results for the OERs obtained from the data in Figure 8.

Catalyst ^[a]^	E_onset_^[b]^ (mV)	E ^[b],[c]^ (mV)	ΔE_onset_ ^[d]^ (mV)	ΔE ^[e]^ (mV)	Tafel (mV dec^−1^)
RuO_2_ [RuCl_3_]	1509 ± 10	1755 ± 7	855 ± 18	1200 ± 14	147 ± 7
RuO_2_ [Cym]	1498 ± 12	--	801 ± 22	--	145 ± 10
RuO_2_ [PPh_3_]	1505 ± 8	1670 ± 10	762 ± 18	1068 ± 15	153 ± 8

[a] Calcined at 900 °C. [b] mV vs. RHE. [c] ΔE_onset_ = (E_OER_ − E_ORR_). [d] ΔE = (E_10−_E_−1_). [e] Tafel plots are displayed in Figure S10.

Recently, the catalyst properties of a RuO_2_−based material containing different oxidation states of Ru, including Ru metal, have been analyzed, displaying Tafel slope values close to ours [59]. On the basis of this result, the next OER mechanism can be proposed for our material [59,60]:

(5) $${\mathrm{OH}}_{\mathrm{aq}}^{-}\;\to {\mathrm{OH}}_{\mathrm{ad}}^{-}\;$$

(6) $${\mathrm{OH}}_{\mathrm{ad}}^{-}\;\to {\mathrm{OH}}_{\mathrm{ad}}^{}+1e^{-}$$

(7) $${\mathrm{OH}}_{\mathrm{ad}}^{}\;\to O_{\mathrm{ad}}^{}+H_{\mathrm{aq}}^{+}+1e^{-}$$

(8) $$O_{\mathrm{ad}}^{}+O_{\mathrm{ad}}^{}\to O_{2\;g}$$

(9) $$H_{\mathrm{aq}}^{+}+{\mathrm{OH}}_{\mathrm{aq}}^{-}\;\to H_{2}O_{\mathrm{aq}}^{}$$

As it has been reported, the Tafel value obtained indicates the rate−determining step; thus, the slope obtained for our materials indicates that reaction (6) will determine the rate of the OER electrocatalytic process [59].

In addition, the stability of the materials was tested by cyclic voltametry. Figure 8E compares the voltamograms registered before and after carrying out 500 cycles in the range of 0.2–0.8 V (vs. Hg/HgO) for RuO_2_ [PPh_3_] at 900 °C. Only a 5.7% intensity decrease was observed between the first and the last cycle, confirming the good stability of this material. Similar graphs for the other materials tested in this work, including commercial RuO_2_, RuO_2_ [RuCl_3_], and RuO_2_ [PPh_3_] calcined at 500 and 1000 °C, are included in Figure S11 in the supplementary information file, which evidences much lower stability for these catalysts with respect to RuO_2_ [PPh_3_] at 900 °C.

#### 3.2.3. Zn/Battery Discharge/Charge

Many technical works have studied catalytic properties towards the ORR and OER processes of the most diverse materials without evaluating their catalytic activity in real systems. Thus, when they are examined by RDE or RRDE, materials with high catalytic activity fail when tested in real batteries, or simply they are not tested. Furthermore, the electrocatalytic activity obtained in RDE and RRDE tests should be completed using the catalysts to prepare air electrodes, which should be incorporated in real operating devices: in our case, in Zn/air batteries [61]. It would demonstrate that the materials under study catalyze both the ORR and OER processes during battery cycling in an alkaline medium [62].

**Figure 9:**
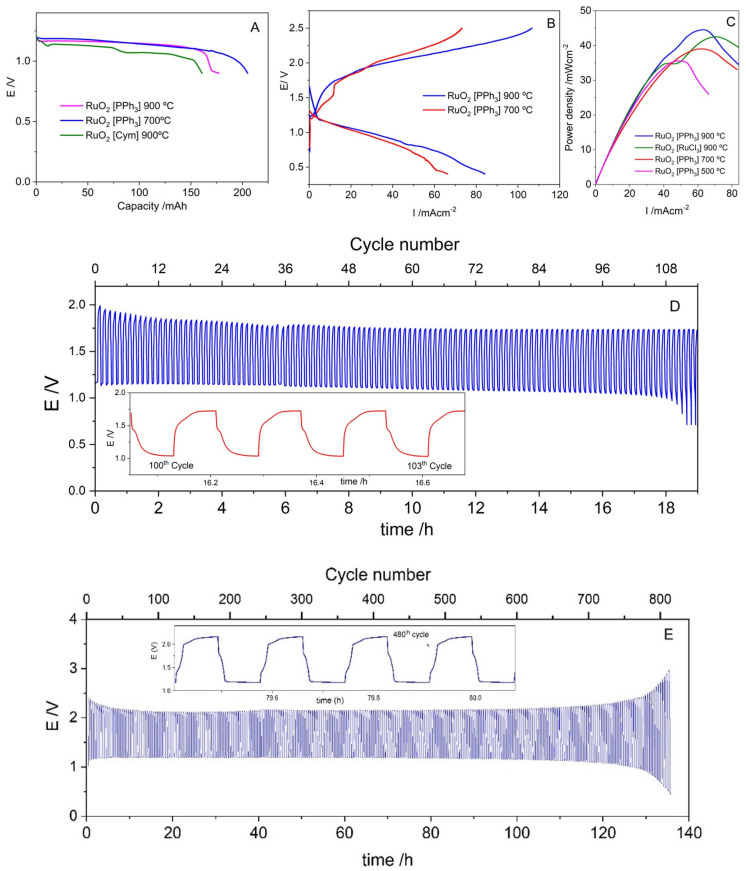
(**A**) Discharge curves of Zn/PVA−KOH/air batteries at −10 mA cm^−2^ utilizing positive electrodes with the different catalysts. (**B**) Polarization curves for the discharge and charge processes of Zn/PVA−KOH/air batteries, with RuO_2_ [PPh_3_] at 900 °C and 700 °C included in the air electrode. (**C**) Power vs. current densities obtained during the polarization tests of the Zn/PVA−KOH/air batteries using air electrodes with different catalysts. (**D**) Discharge/charge cycles of a Zn/PVA−KOH/air battery, adding RuO_2_ [PPh_3_] 900 °C as a catalyst in the air electrode. (**E**) Discharge/charge cycles of a flooded Zn/air battery in ~1mL 8M KOH solution, adding RuO_2_ [PPh_3_] 900 °C as the catalyst in the air electrode.

In this work, the RuO_2_−based catalysts were also deposited onto air electrodes and tested in Zn/PVA−KOH/air batteries in order to demonstrate their applicability as bifunctional catalysts in a real Zn/air battery. As seen in Figure 9A, the highest discharge capacity was obtained for RuO_2_ [PPh_3_] 900 °C: 205 mA h, followed by RuO_2_ [PPh_3_] 700 °C: 178 mA h, and RuO_2_ [Cym]. Hence, taking into account the catalyst mass, 0.015 ± 0.005 g, the specific capacities can be calculated with respect to the catalyst amount (10.5 A h g^−1^, 11.5 A h g^−1^, and 8.1 A h g^−1^, respectively) and considering the catalyst and carbon (5.2, 5.4, and 4.0 A h g^−1^). Furthermore, energies of 2.1 W h and 1.8 W h were obtained for RuO_2_ [PPh_3_] 900 °C and 700 °C, respectively, and contemplating an average voltage value of 1.14 V during the discharge, specific energies of 12.0 and 13.1 kW h kg^−1^ (considering the catalyst mass) and 5.9 and 6.2 kW h kg^−1^ (considering the catalyst and catalyst + carbon mass) resulted, respectively. These values widely exceed those of the capacity and specific energy values previously published by different authors, which reported values lower than 0.8 kW h kg^−1^ [63,64,65]. In addition to this, polarization measurements were carried out for the Zn/PVA−KOH/air batteries utilizing the air electrodes, which were made from different RuO_2_−based catalysts. Figure 9B compares the potential vs. current density discharge and charge curves for RuO_2_ [PPh_3_] 700 °C and 900 °C, and, once again, the latter shows the best results. Besides, Figure 9C displays the power vs. current densities obtained, where RuO_2_ [PPh_3_] 900 °C saw the maximum power density value of ~45 mW cm^−2^ and a specific power value of 4.4 kW kg^−1^ cm^−2^. Finally, we carried out long−term cycling tests on an all−solid battery, Zn/PVA−KOH/air, and a flooded Zn/air version, using RuO_2_ [PPh_3_] 900 °C as the catalyst in the air electrodes. Applying a specific current of 41.6 mA g^−1^ and specific discharge and charge capacities of 6.94 A h g^−1^ (on the basis of the catalytic mass) to the Zn/PVA−KOH/air, more than 105 cycles were reached with a discharge potential higher than 1.0 V and a stable charge potential of 1.6 V (Figure 9D). The inset of this figure shows four magnified consecutive cycles, where a potential gap of 0.69 V between the charge and discharge was maintained beyond 100 cycles, confirming excellent energy efficiency. Besides, a Zn/8M KOH/air battery has been tested using a specific current of 10.7 A g^−1^ and specific charge and discharge 0.89 A h g^−1^ (in basis of the catalytic mass). More than 840 cycles were carried out when RuO_2_ [PPh_3_] 900 °C was added as catalyst in the air electrode (Figure 9E). Moreover, the superior catalytic properties of this material in both charge and discharge processes is demonstrated in Figure S12, where two Zn/air batteries using RuO_2_ [PPh_3_] 900 °C and commercial RuO_2_ in the air electrode are compared.

## 4. Discussion

The results presented in this work point to RuO_2_ [PPh_3_] 900 °C as the catalyst that displays the best properties in terms of a high ORR current density, a lower ΔE_onset_, an n value for ORRs close to 4, as well as the best current density and stable behavior for OERs. Besides, when this material is included in the air electrode of all−solid and flooded Zn/air batteries, the highest power density, number of cycles, and discharge capacity values were found. On the other hand, good catalytic properties were also observed for the material obtained from the same precursor but calcined at 700 °C.

The XRD, EDX, and SEM measurements confirmed the presence of phosphorous (probably forming RuP_3_O_9_) and Ru metal inside the materials derived from [(RuCl_2_(PPh_3_)_3_]. However, neither phosphorous nor Ru metal have been detected in the materials obtained by calcination from [RuCl_3_∙xH_2_O] and [RuCl_2_(p−cymene)]_2_. This fact, together with the best electrocatalytic properties obtained for RuO_2_ [PPh_3_] 900 °C, points to a synergetic effect of Ru/RuO_2_, as has already been reported [66,67]. Besides, it cannot be ruled out that the improvement in the catalytic activity toward the ORR and OER processes is influenced by the existence of RuP_3_O_9_. Ruthenium phosphide has been previously used as an ORR catalyst in acid solutions [19]. In any case, the participation of phosphorous inside the catalyst material has been revealed to be fundamental for maintaining Ru metal.

It has to be highlighted that the calcination process for [RuCl_2_(PPh_3_)_3_] is very different from that of the other precursors. Besides the existence of phosphorous, the TGA analysis, XPS spectra, and the XRD patterns [30] demonstrated the presence of Ru metal during the calcination process from the lower temperature, which was not observed for the calcination of [RuCl_3_∙xH_2_O] and [RuCl_2_(p−cymene)]_2_. It is known that phosphines are strong reductor groups, and they have to reduce the Ru(II) of the starting compound to Ru(0), initially inhibiting ruthenium oxidation despite the air atmosphere. However, the amount of Ru metal diminishes with temperature, while increasing the RuO_2_. Thus, the calcination of [RuCl_2_(PPh_3_)_3_] would produce a hybrid material with the composition xRu(1−x)RuO_2_, where x decreases with temperature.

Furthermore, the good catalyst properties obtained for the RuO_2_ [PPh_3_] materials could be justified by the amount of Ru metal remaining in the catalyst, together with RuO_2_ [16,57]. However, as has been explained, the quantity of Ru metal inside the material decreases with calcination temperature; from here, those materials calcined at 500 and 700 °C should better catalyze the OER and/or ORR processes than the material obtained at 900 °C. Nevertheless, the results obtained in this work contradict this statement, pointing to the synergetic contribution of Ru/RuO_2_ and RuP_3_O_9_ to electrocatalysis. As can be seen in Figure 4B, the XRD pattern of the RuO_2_ [PPh_3_] calcined at 500 °C shows very intense peaks for Ru metal and lower peaks for RuO_2_, demonstrating a higher amount of Ru metal in this material with respect to those obtained at 700 °C and 900 °C [30]; however, the catalyst properties of the material obtained at 500 °C for the ORR and OER are very poor, as has been evidenced in this work. Besides, it should be noted that no peaks for RuP_3_O_9_ were observed in the XRD pattern, which instead appeared for the RuO_2_ [PPh_3_] calcined at 700 °C and 900 °C. On the contrary, calcination at higher temperatures, such as 1000 °C, eliminates all Ru metal and RuP_3_O_9_ residues, providing a material composed only by RuO_2_, which evidenced worse catalytic properties than the RuO_2_ [PPh_3_] calcined at 700 °C and 900 °C, but similar to RuO_2_ [RuCl_3_] and RuO_2_ [Cym], revealing the importance of Ru metal and RuP_3_O_9_ to improve the catalytic activity of RuO_2_.

## 5. Conclusions

In this work, the synthesis of RuO_2_−based catalysts with excellent electrocatalytic properties for ORRs and OERs was successfully completed. Singular materials were prepared through a thermal calcination process using a selection of different Ru−containing reagents: [RuCl_2_(PPh_3_)_3_], [RuCl_3_∙xH_2_O], and [RuCl_2_(p−cymene)]_2_. The results obtained demonstrate the superior electrocatalytic properties of the RuO_2_ [PPh_3_] calcined at 900 °C for ORRs and OERs, with respect to the other RuO_2_−based materials analyzed. This fact was corroborated via the LSV analysis, with ΔE_onset_ = 762 mV, and also when the material was incorporated into air electrode of the Zn/PVA−KOH/air batteries. A specific discharge capacity of 10.5 A h g^−1^ at 250 mA g^−1^, a power density of 4.4 kW kg^−1^ cm^−2^, and more than 100 discharge/charge cycles with potential values higher than 1.0 V during discharge resulted. Besides, more than 800 discharge/charge cycles were reached for a flooded alkaline Zn/air battery. This improvement in the electrocatalytic properties may be justified by the synergetic effect of the RuO_2_, Ru metal, and RuP_3_O_9_ present in RuO_2_ [PPh_3_] calcined at temperatures between 700 °C and 900 °C. However, this affirmation should be confirmed by carrying out additional measurements and DFT analyses.

## Figures and Tables

**Table 1:** Electrochemical parameters obtained from the data in Figure 5.

Compound	E_onset_^[a]^ (mV)	E ^[a],[b]^ (mV)	n_KL_^[c]^	n_RRDE_^[c]^	Tafel ^[d]^ (mV dec^−1^)
RuO_2_ [RuCl_3_]	654 ± 8	555 ± 7	3.8	3.4	188 ± 1
RuO_2_ [Cym]	697 ± 8	462 ± 10	2.6	2.6	82 ± 3
RuO_2_[PPh_3_] 500 °C	677 ± 20	522 ± 15	2.6	3.1	155 ± 3
RuO_2_[PPh_3_] 700 °C	714 ± 1	514 ± 7	2.8	3.8	106 ± 9
RuO_2_[PPh_3_] 900 °C	753 ± 10	602 ± 5	3.6	3.6	106 ± 11

[a] (mV vs. RHE). [b] E at −1mA cm^−2^. [c] Values at 0.265 V vs. RHE. [d] Tafel slopes depicted in Figure S9.

## Data Availability

Not applicable.

## Supplementary Materials

The following are available online at https://www.mdpi.com/article/10.3390/nano13010115/s1. The supplementary information file includes additional details about the electrochemical experiments. References [36,51,67] are cited in the supplementary materials.
