# Supplementary material for: Bifunctional P-Containing RuO2 Catalysts Prepared from Surplus Ru Co-Ordination Complexes and Applied to Zn/Air Batteries
Source: Nanomaterials (Basel). 2022 Dec 26;13(1):115. doi: 10.3390/nano13010115 (PMC9824568; doi:10.3390/nano13010115)
Supplement: Supplementary file 1 [file nanomaterials-13-00115-s001.zip › nanomaterials-2037517-supplementary.pdf]

Supplementary Materials

Bifunctional P-containing RuO_2 Catalysts Prepared from Surplus Ru Co-ordination Complexes and Applied to Zn/Air Batteries

Sebastián Lorca [1], Javier Torres [1], José L. Serrano [2], José Pérez [2], José Abad [1], Florencio Santos [1] and Antonio J. Fernández Romero [1*]

[1] Grupo de Materiales Avanzados para la Producción y Almacenamiento de Energía, Universidad Politécnica de Cartagena, Aulario II, Campus de Alfonso XIII, 30203 Cartagena, Spain; sebastian.lorca@edu.upct.es (S.L.); javiertorresuniv@gmail.com (J.T.); jose.abad@upct.es (J.A.); florencio.santos@upct.es (F.S.)
[2] Departamento de Ingeniería Química, Área de Química Inorgánica. Universidad Politécnica de Cartagena. Plaza del Hospital 1, 30203 Cartagena, Spain; jose.serrano@upct.es (J.L.S.); jose.pperez@upct.es (J.P.)
* Correspondence: antonioj.fernandez@upct.es

1. Structural characterization

1.1. EDX and SEM Measurements

Table S1. Ru and P ratios for [$RuCl_2$-(PPH_3)$_3$] obtained at different temperatures.

Sample [RuO_2 [PPh_3]	Ru (%m norm.)	P (%m norm.)	P/Ru
**500 °C**	35.10	16.58	0.47
**700 °C**	41.23	15.73	0.38
**900 °C**	57.64	1,61	0.03

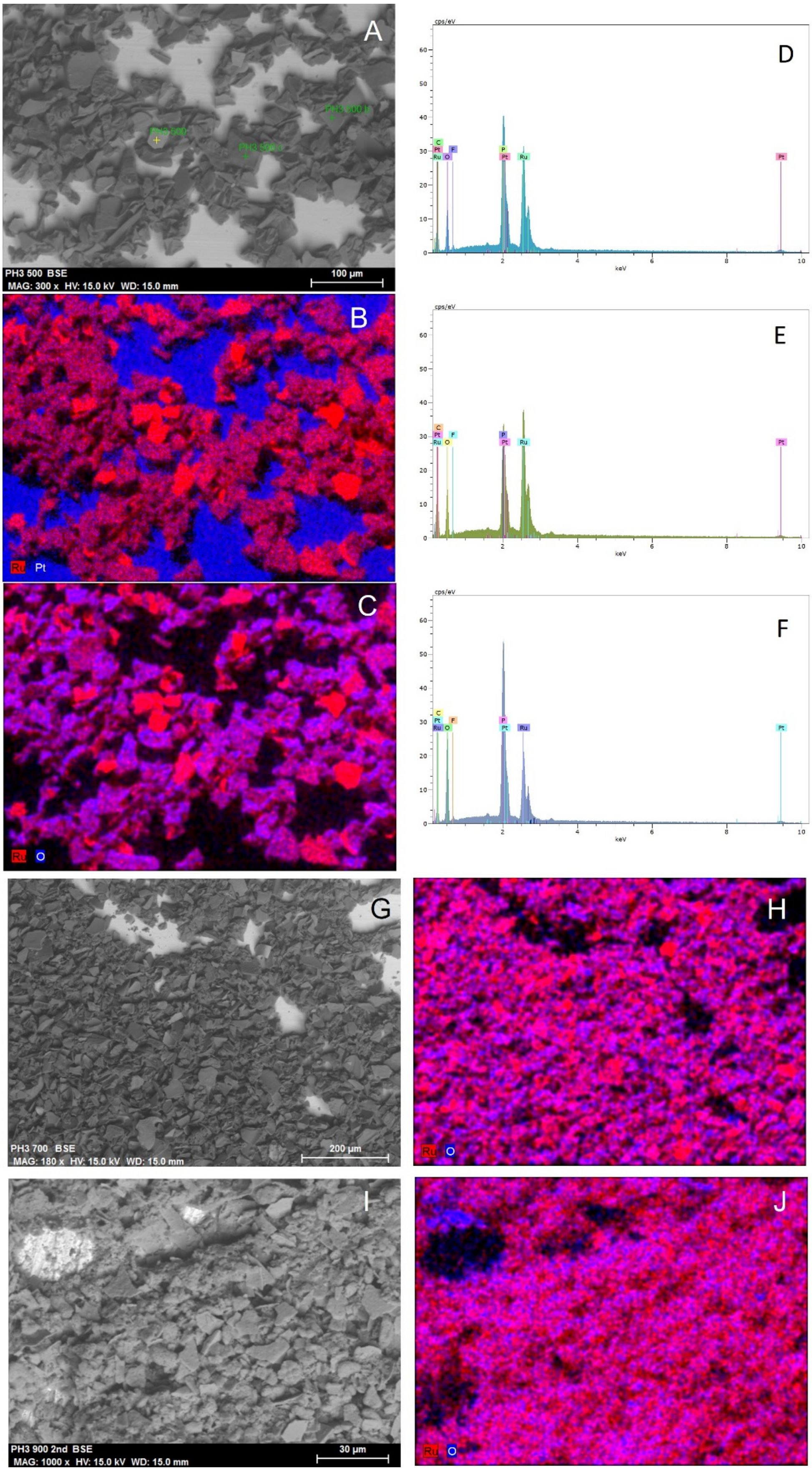

Figure S1. SEM images of RuO_2 [PPh_3] obtained at 500 °C (**A**), 700 °C (**G**) and 900 °C (**I**). EDX mapping of RuO_2 [PPh_3] 500 ((**B**): Ru, Pt and (**C**): Ru, O), 700 (**H**) and 900 (**J**). (**D**–**F**) EDX for the marked points on (**A**).

1.2. TGA

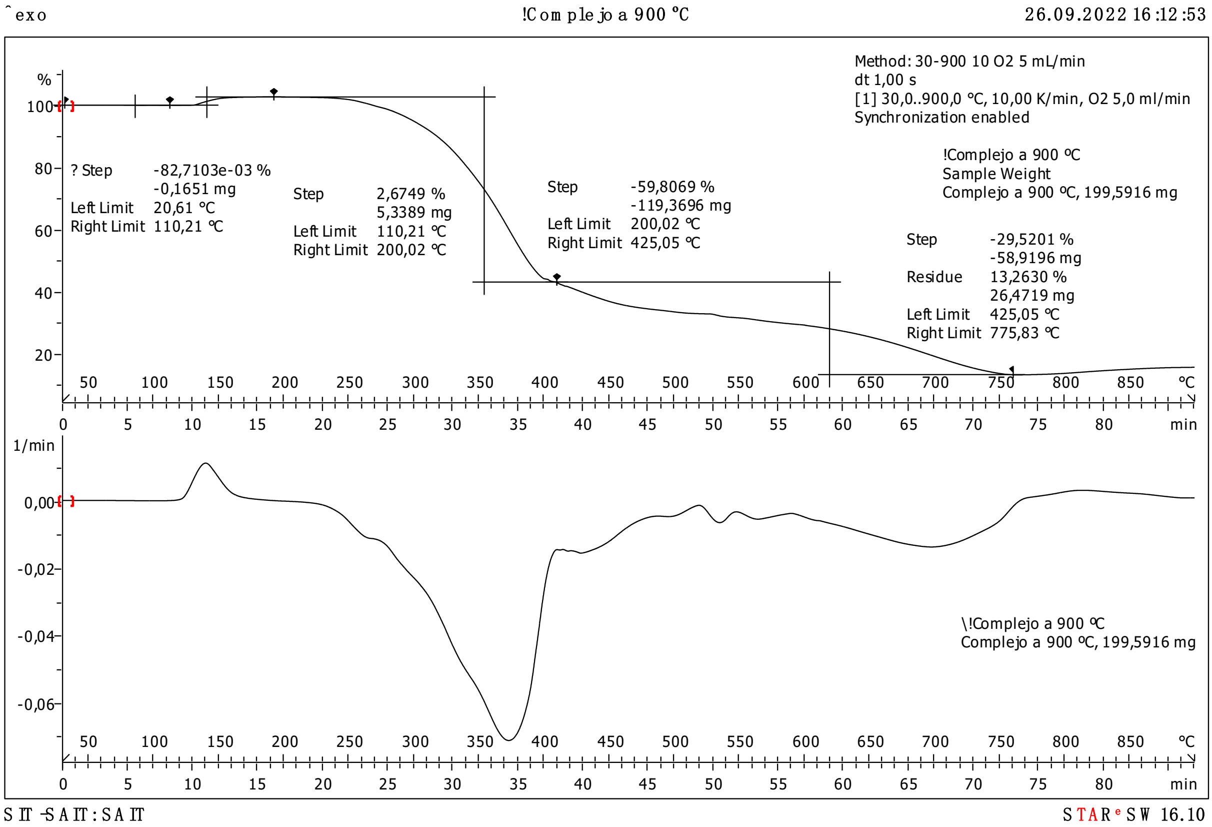

Figure S2. TG and DTG curves registered for [$RuCl_2$-(PPh_3)$_3$].

1.3. XPS

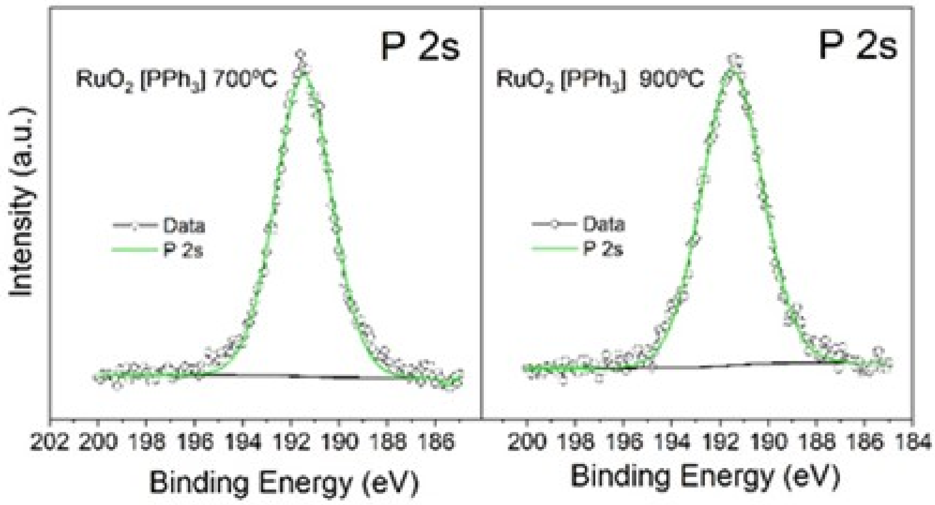

Figure S3. XPS of the P2s region.

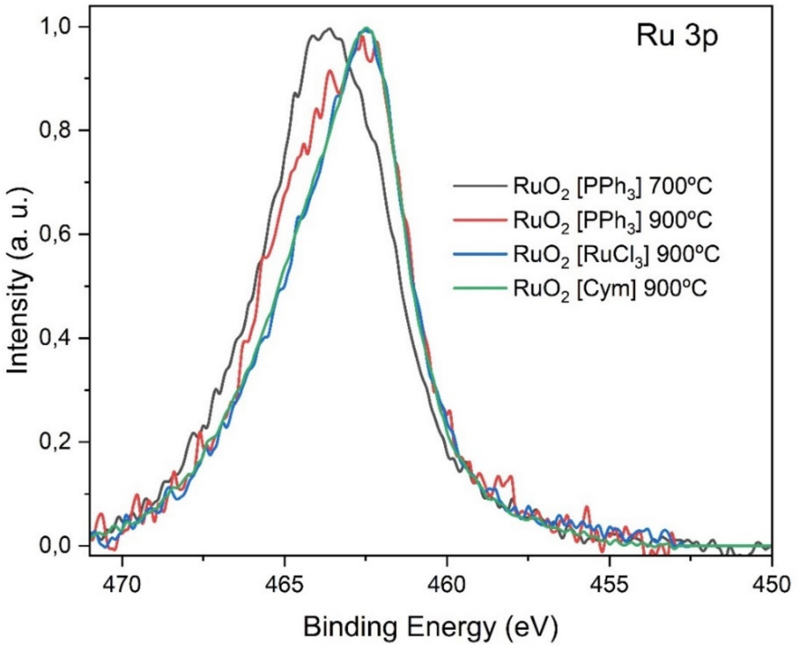

Figure S4. XPS of the Ru3p region.

Table S2. Fitting parameters of the XPS peaks deconvolutions.

Sample	Peak	Asigned	BE (eV)	FWHM (eV)	Area	Lineshape
RuO_2 [PPH3] 700 °C	Ru $3d_{5/2}$	Ru (IV)	280.9	0.5	1438	Asymetric GL
	Ru $3d_{5/2}$	Satellite	282.5	0.8	719	Asymetric GL
	Ru $3d_{5/2}$	Ru (III)	282.8	1.9	2841	Asymetric GL
	C 1s		284.8	1.7	944	G(70)L(30)
	Ru $3d_{3/2}$	Ru (IV)	285.1	1.1	963	Asymetric GL
	Ru $3d_{3/2}$	Satellite	286.7	1.4	482	Asymetric GL
	Ru $3d_{3/2}$	Ru (III)	287.0	2.1	1903	Asymetric GL
RuO_2 [PPH3] 900 °C	Ru $3d_{5/2}$	Ru (0)	280.2	0.4	167	Asymetric GL
	Ru $3d_{5/2}$	Ru (IV)	280.8	0.6	1354	Asymetric GL
	Ru $3d_{5/2}$	Satellite	282.2	1.3	677	Asymetric GL
	Ru $3d_{5/2}$	Ru (III)	283.3	1.5	731	Asymetric GL
	Ru $3d_{5/2}$	Ru (0)	284.5	0.4	112	Asymetric GL
	C 1s		284.7	1.8	763	G(70)L(30)
	Ru $3d_{3/2}$	Ru (IV)	285.0	1.0	907	Asymetric GL
	Ru $3d_{3/2}$	Satellite	286.4	1.3	454	Asymetric GL
	Ru $3d_{3/2}$	Ru (III)	287.5	1.7	490	Asymetric GL
RuO_2 [RuCl3] 900 °C	Ru $3d_{5/2}$	Ru (IV)	280.2	0.5	5739	Asymetric GL
	Ru $3d_{5/2}$	Satellite	282.7	1.4	2851	Asymetric GL
	C 1s		284.7	1.6	1429	G(70)L(30)
	Ru $3d_{3/2}$	Ru (IV)	285.0	1.0	3845	Asymetric GL
	Ru $3d_{3/2}$	Satellite	286.9	1.6	1910	Asymetric GL
RuO_2 [Cym] 900 °C	Ru $3d_{5/2}$	Ru (IV)	280.8	0.5	5598	Asymetric GL
	Ru $3d_{5/2}$	Satellite	282.7	1.3	2645	Asymetric GL
	C 1s		284.7	2.4	1447	G(70)L(30)
	Ru $3d_{3/2}$	Ru (IV)	285.0	1.0	3751	Asymetric GL
	Ru $3d_{3/2}$	Satellite	286.8	1.6	1772	Asymetric GL
RuO_2 [PPH3] 700 °C	O 1s	Ru-O	529.6	0.8	334	Asymetric GL
	O 1s	P-O-P	531.3	1.5	1809	G(70)L(30)
	O 1s	Satellite	531.5	1.9	161	Asymetric GL
	O 1s	Ru-O-P	533.0	1.8	1551	G(70)L(30)
RuO_2 [PPH3] 900 °C	O 1s	Ru-O	529.4	0.7	234	Asymetric GL
	O 1s	P-O-P	531.2	1.6	1090	G(70)L(30)
	O 1s	Satellite	531.3	1.7	112	Asymetric GL
	O 1s	Ru-O-P	532.6	2.0	1973	G(70)L(30)
	O 1s	O2/H2O	535.8	1.9	97	G(70)L(30)
RuO_2 [RuCl3] 900 °C	O 1s	Ru-O	529.4	0.7	1008	Asymetric GL
	O 1s	screening	530.3	1.4	680	G(70)L(30)
	O 1s	Satellite	531.3	1.6	484	Asymetric GL
	O 1s	C-O	532.4	2.0	387	G(70)L(30)
RuO_2 [Cym] 900 °C	O 1s	Ru-O	529.4	0.7	981	Asymetric GL
	O 1s	screening	530.3	1.4	654	G(70)L(30)
	O 1s	Satellite	531.2	1.7	471	Asymetric GL
	O 1s	C-O	532.5	2.1	406	G(70)L(30)
RuO_2 [PPH3] 700 °C	P 2s	$(PO3)^-$	191.5	2.7	923	G(70)L(30)
RuO_2 [PPH3] 900 °C	P 2s	$(PO3)^-$	191.5	3.0	736	G(70)L(30)

:

2. Electrochemical Characterization

The electro-chemical performance tests were conducted by using a Biologic VSP Modular 5 channels potentiostat/galvanostat electrochemical workstation coupled with a rotating disk-ring electrode system RRDE-3A (Als Co.Ltd.) consisting of a (glassy carbon) GC disk-Pt-ring as working electrode. Besides, a reference electrode of Hg/HgO and a GC bar were used in a three electrode cell.

Voltage values have been converted to a reversible hydrogen electrode (RHE) scale according to the equation:

$$E_{RHE} = E_{Hg/HgO} + 0.059 \times pH + 0.098 \quad \text{(S1)}$$

The electrochemical and hydrodynamic properties of electrocatalyst are evaluated using the Koutecky-Levich expression:

$$\frac{1}{I_D} = \frac{1}{I_K} + \frac{1}{I_L} = \frac{1}{I_K} + \frac{1}{0.62nFAc_{O_2}^{*}D_0^{\frac{2}{3}}v^{-\frac{1}{6}}\omega^{\frac{1}{2}}} \quad \text{(S2)}$$

where n is the electron transfer number, F is the Faraday constant, $D_{O_2} = 1.93\times10^{-5}$ $cm^2\ s^{-1}$ is the diffusion coefficient of O_2 in the electrolyte, $\nu = 1.09\times10^{-2}$ $cm^2\ s^{-1}$ is the kinematic viscosity, and $c_{O_2}^{*} = 0.126 * 10^{-6}$mol cm^{-3} is the bulk concentration of O_2 in the electrolyte using pure O_2.

Besides, due to the controversy on use KL method, the electron transfer number and the percentage ratio of HO_2^- on the electrode potential was tested by the RRDE method based on Equations (S3) and (S4) [1]

$$n = \frac{4I_d}{Id + (\frac{I_r}{N})} \quad \text{(S3)}$$

$$H_2O_2\ (\%) = 200\frac{I_r/N}{I_d + (\frac{I_r}{N})} \quad \text{(S4)}$$

where I_d is the disk current, I_r is the ring current, and N is the geometric factor of the RRDE known more frequently as current collection efficiency of the Pt ring, which was determined to be 42.4 [2,3].

Tafel plots were obtained by representing E vs. Log(I), following the equations:

$$E = a + b\log(I) \quad \text{(S5)}$$

where I is the current density and b is the Tafel slope.

Tafel slope values included in the Table I and II correspond to the average of three RRDE rotation speeds for two different LSV measures.

On the other hand, onset values for ORR and OER were calculated from the initial point where the Tafel slope is separated from the experimental curve.

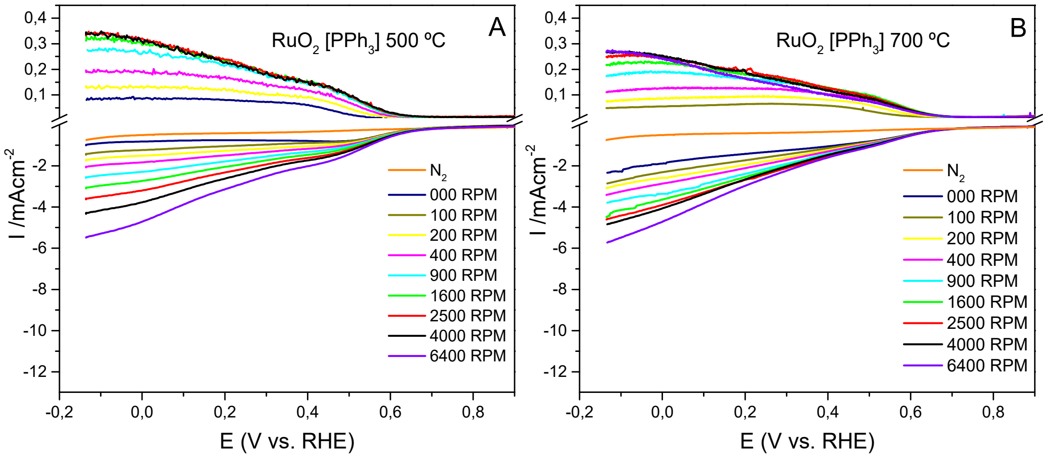

Figure S5. LSV curves of: A) RuO_2 [PPh_3] calcined at 500 ºC; b) RuO_2 [PPh_3] calcined at 700 ºC, obtained using a RRDE in O_2 and N_2 saturated 0.1 KOH. Rotation rates values are included in each graph.

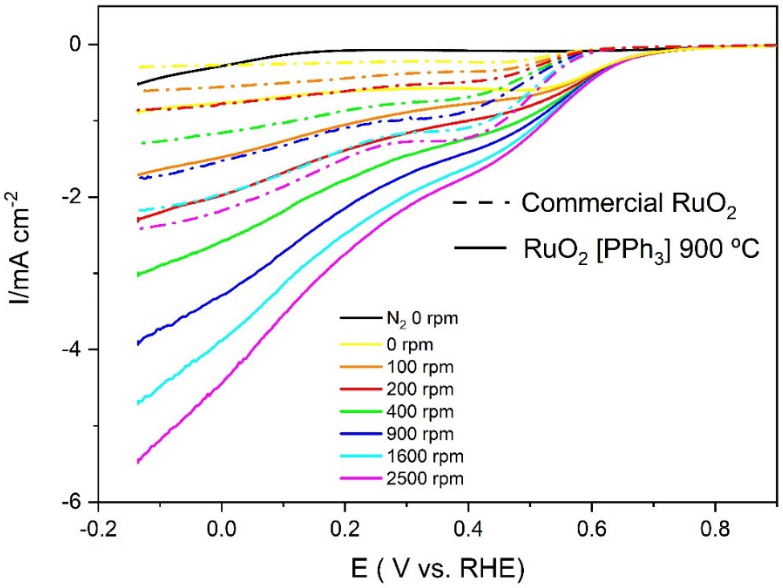

Figure S6. LSV curves for RuO_2 [PPh_3] 900 ºC and Commercial RuO_2 purchased from Merck.

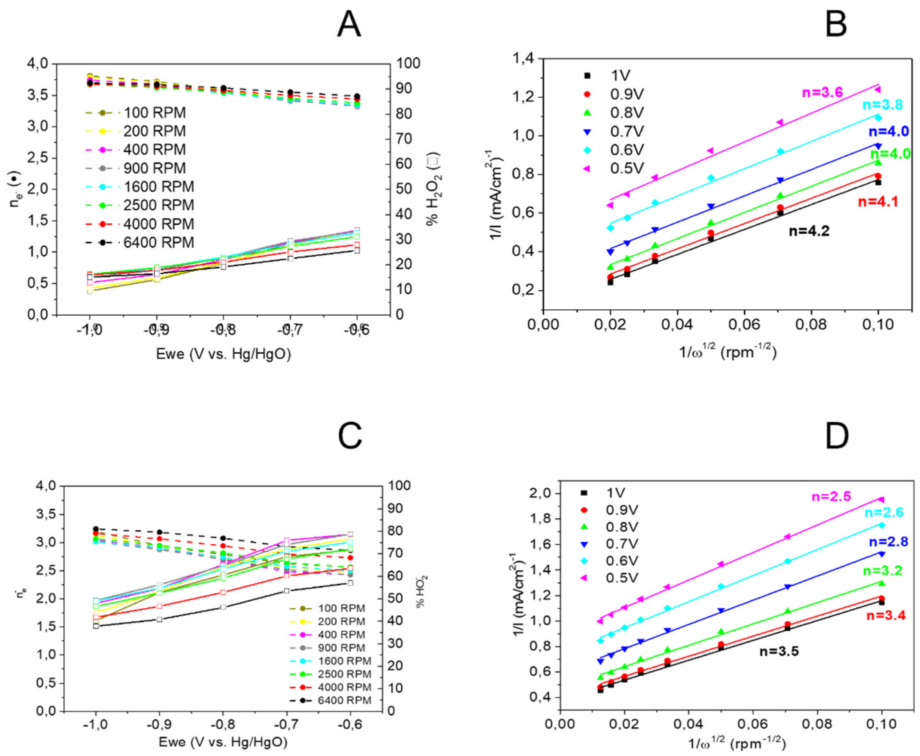

Figure S7. (**A**,**C**) Dependence of the electron transfer number and HO_2^- values with the electrode potential at different rotation rates from data in Figure 5, using Equation (S3) and (S4). (**B**,**D**) KL plots. (**A**,**B**) for RuO_2 [$RuCl_3$] and RuO_2 [Cym] calcined at 900 ºC.

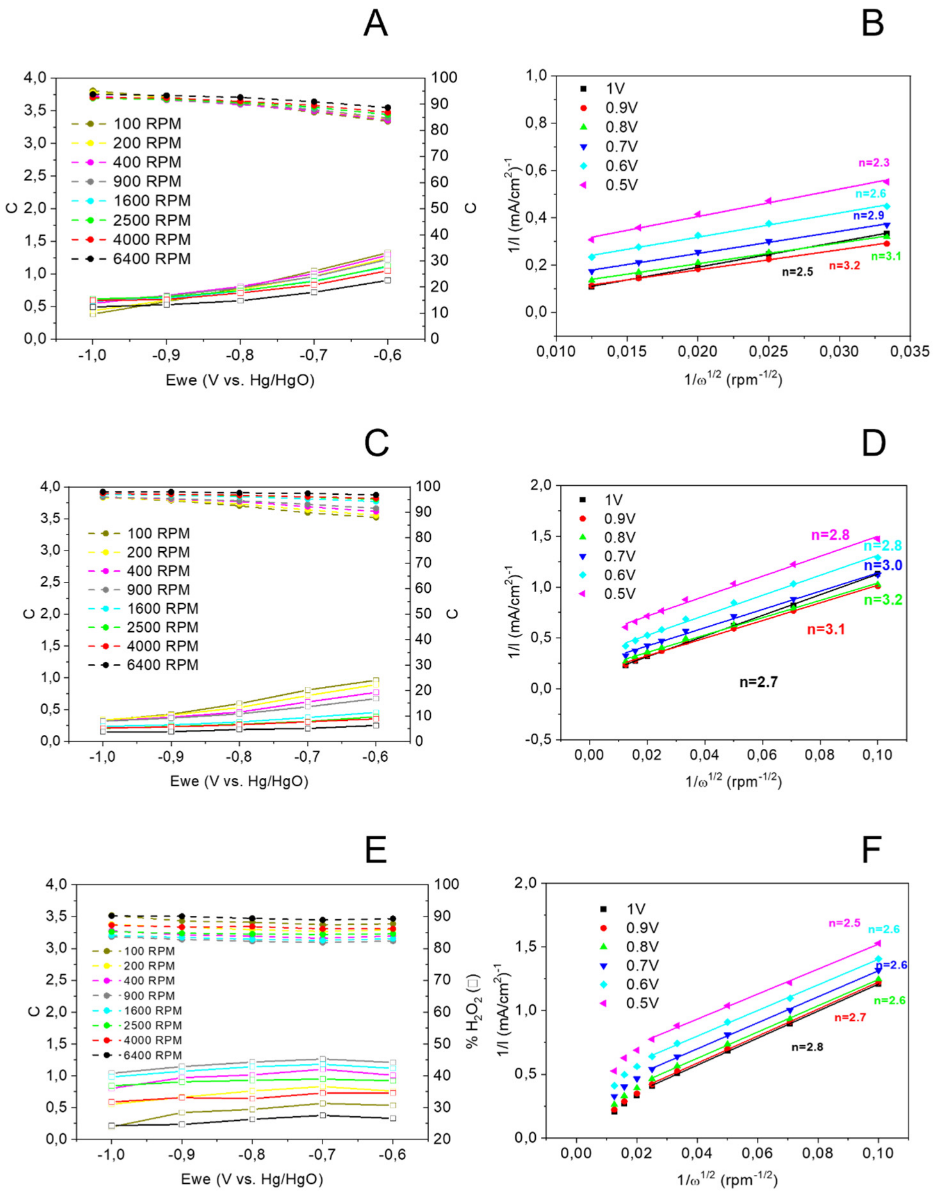

Figure S8. (**A**,**C**,**E**) Dependence of the electron transfer number and HO_2^- values with the electrode potential at different rotation rates from data in Figure 5, using Equations (S3) and (S4). (**B**,**D**,**F**) KL plots. (**A**,**B**) for RuO_2 [PPh_3] at 900 °C; (**C**,**D**) RuO_2 [PPh_3] at 700 °C and (**E**,**F**) RuO_2 [PPh_3] at 500 ºC.

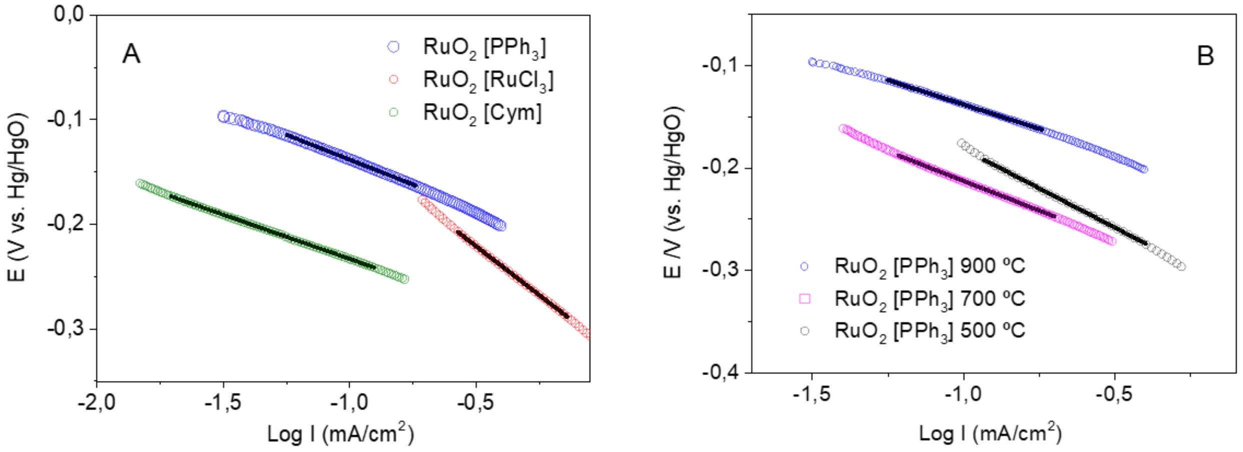

Figure S9. Tafel plots for ORR of the RuO2-based catalysts. (**A**) RuO2 [PPh3], RuO2 [RuCl3] and RuO2 [Cym] calcined at 900 °C and (**B**) the three RuO2 [PPh3] obtained at 500, 700 and 900 ºC.

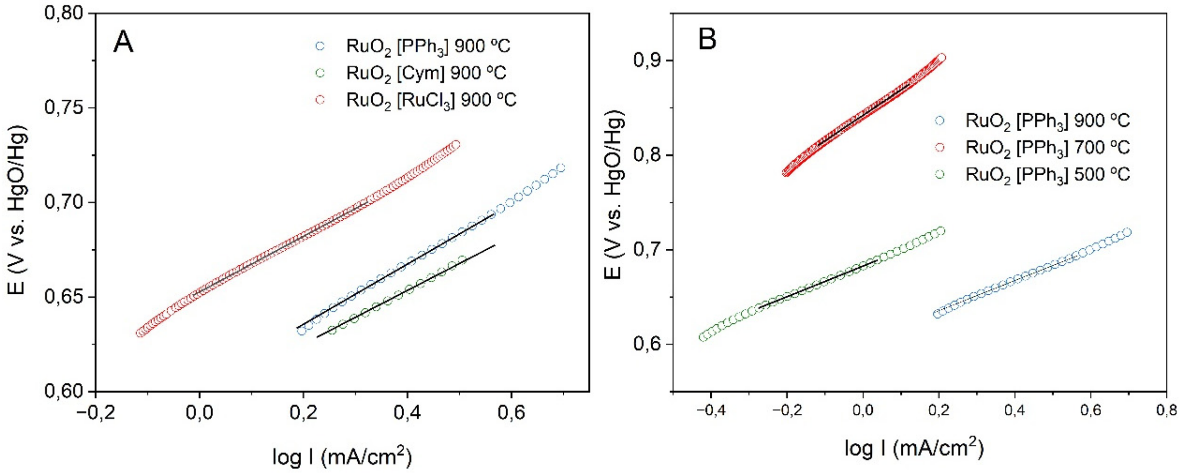

Figure S10. Tafel plots for OER of the RuO2-based catalysts. (**A**) RuO2 [PPh3], RuO2 [RuCl3] and RuO2 [Cym] calcined at 900 °C and (**B**) the three RuO2 [PPh3] obtained at 500, 700 and 900 °C.

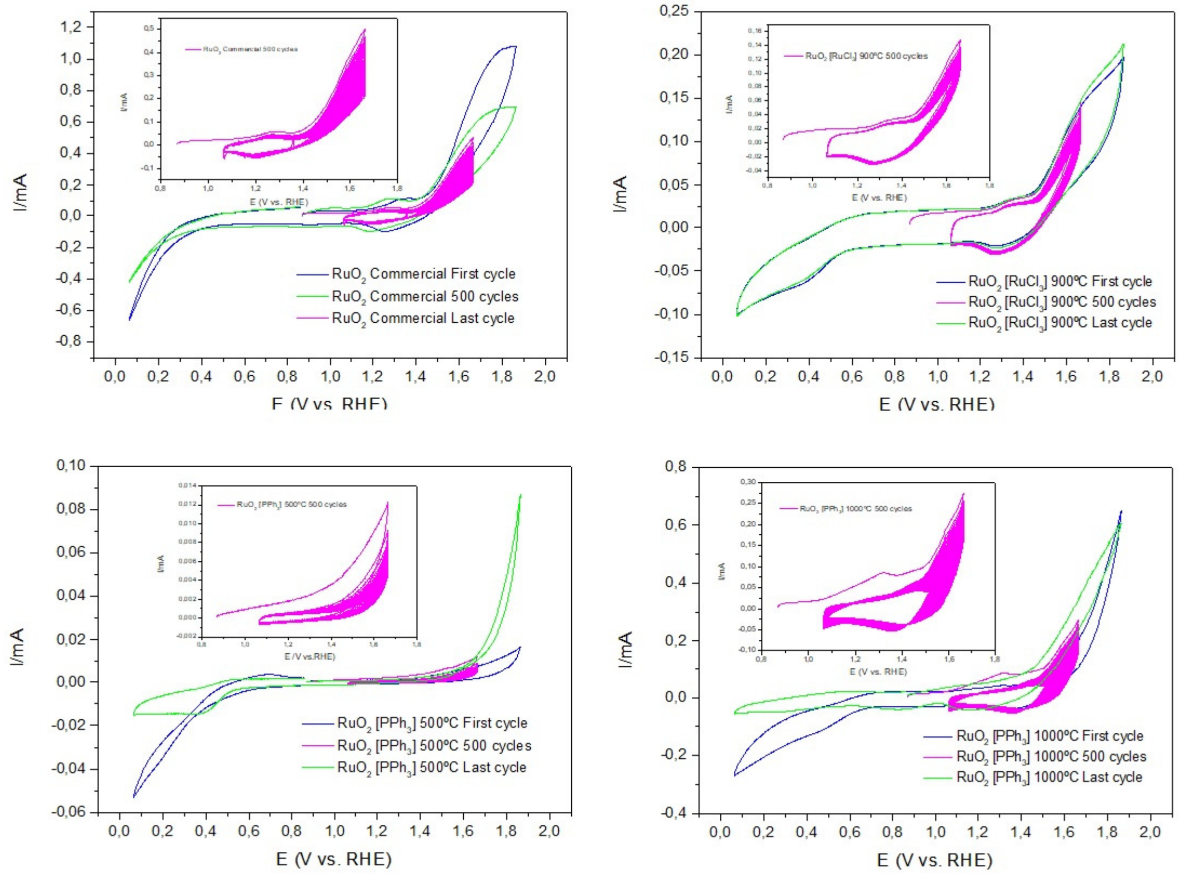

Figure S11. Stability CV plots obtained for (**A**) Commercial RuO_2 RuO_2 , (**B**) RuO_2 [$RuCl_3$], (**C**) RuO_2 [PPh_3] 500 °C and (**D**) RuO_2 [PPh_3] 1000 °C.

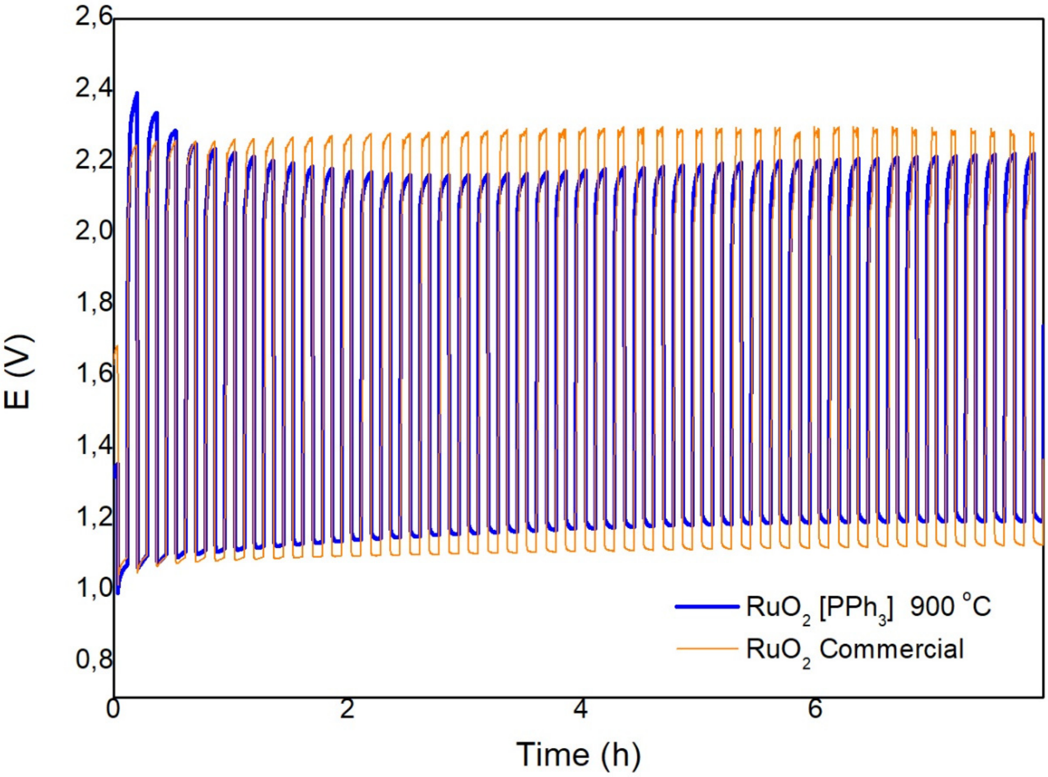

Figure S12. Discharge/Charge cycles of a flooded Zn/air battery in ~1mL 8M KOH solution, adding RuO2 [PPh3] 900 °C and Commercial RuO_2.

References

1. Du, C.; Tan, Q.; Yin, G.; Zhang, J. Rotating Ring-Disk Electrode Method. In Rotating Electrode Methods and Oxygen Reduction Electrocatalysts; Elsevier: Amsterdam, The Netherlands, 2014; pp. 171–198. https://doi.org/10.1016/B978-0-444-63278-4.00005-7.
2. Dalton, F. Historical Origins of the Rotating Ring-Disk Electrode. *Electrochem. Soc. Interface* **2016**, *25*, 50–59.
3. Garsany, Y.; Ge, J.; St-Pierre, J.; Rocheleau, R.; Swider-Lyons, K. ORR Measurements Reproducibility Using a RRDE. *ECS Trans.* **2013**, *58*, 1233–1241. https://doi.org/10.1149/05801.1233ecst.
